# Domain-driven models yield better predictions at lower cost than reservoir computers in Lorenz systems

**DOI:** 10.1098/rsta.2020.0246

**Published:** 2021-02-15

**Authors:** Ryan Pyle, Nikola Jovanovic, Devika Subramanian, Krishna V. Palem, Ankit B. Patel

**Affiliations:** Baylor College of Medicine, Rice University Houston, TX, USA

**Keywords:** reservoir computing, weather prediction, dynamical systems, echo state networks, inexact computing

## Abstract

Recent advances in computing algorithms and hardware have rekindled interest in developing high-accuracy, low-cost *surrogate* models for simulating physical systems. The idea is to replace expensive numerical integration of complex coupled partial differential equations at fine time scales performed on supercomputers, with machine-learned surrogates that efficiently and accurately forecast future system states using data sampled from the underlying system. One particularly popular technique being explored within the weather and climate modelling community is the *echo state network* (ESN), an attractive alternative to other well-known deep learning architectures. Using the classical Lorenz 63 system, and the three tier multi-scale Lorenz 96 system (Thornes T, Duben P, Palmer T. 2017 *Q. J. R. Meteorol. Soc.* **143**, 897–908. (doi:10.1002/qj.2974)) as benchmarks, we realize that previously studied state-of-the-art ESNs operate in two distinct regimes, corresponding to low and high spectral radius (LSR/HSR) for the sparse, randomly generated, reservoir recurrence matrix. Using knowledge of the mathematical structure of the Lorenz systems along with systematic ablation and hyperparameter sensitivity analyses, we show that state-of-the-art LSR-ESNs reduce to a polynomial regression model which we call Domain-Driven Regularized Regression (D2R2). Interestingly, D2R2 is a generalization of the well-known SINDy algorithm (Brunton SL, Proctor JL, Kutz JN. 2016 *Proc. Natl Acad. Sci. USA* **113**, 3932–3937. (doi:10.1073/pnas.1517384113)). We also show experimentally that LSR-ESNs (Chattopadhyay A, Hassanzadeh P, Subramanian D. 2019 (http://arxiv.org/abs/1906.08829)) outperform HSR ESNs (Pathak J, Hunt B, Girvan M, Lu Z, Ott E. 2018 *Phys. Rev. Lett.* **120**, 024102. (doi:10.1103/PhysRevLett.120.024102)) while D2R2 dominates both approaches. A significant goal in constructing surrogates is to cope with barriers to scaling in weather prediction and simulation of dynamical systems that are imposed by time and energy consumption in supercomputers. *Inexact computing* has emerged as a novel approach to helping with scaling. In this paper, we evaluate the performance of three models (LSR-ESN, HSR-ESN and D2R2) by varying the precision or word size of the computation as our inexactness-controlling parameter. For precisions of 64, 32 and 16 bits, we show that, surprisingly, the least expensive D2R2 method yields the most robust results and the greatest savings compared to ESNs. Specifically, D2R2 achieves 68 × in computational savings, with an additional 2 × if precision reductions are also employed, outperforming ESN variants by a large margin.

This article is part of the theme issue ‘Machine learning for weather and climate modelling’.

## 1. Introduction

Despite impressive advances in hardware and algorithms, modelling many real-world physical systems (such as climate and weather) has proven to be computationally intractable at high spatial resolution, such as a 100 by 100 metre grid. This is due in part to the extreme computational requirements of (a) numerically integrating coupled, high-dimensional, nonlinear partial differential equations for subsystems exhibiting multiple temporal and spatial scales, (b) insufficient data or unknown parameters characterizing sub-processes, and, in some cases, (c) no knowledge of the governing subsystem of equations. In the context of weather prediction, chaotic dynamical systems such as Lorenz 96 [1] have served as an important benchmark, providing the larger community with a ‘toy’ system that enables fast experimentation while still exhibiting the most important physical phenomena that drive computational cost. Motivated by the reasons above and spurred on by the availability of ‘toy’ models, there has been a recent surge in studies of machine learning models [2–7] which produce accurate predictions of the system while requiring less computation or data. The success of largely data-driven deep learning models yielding excellent results on difficult perceptual and prediction tasks such as image classification, speech recognition, medical outcome prediction and handwriting classification have inspired this trend. In addition, machine learning (ML) models offer the ability to learn dynamics directly from (massive quantities of) observational data, and the ability to, when combined with principled causal experimentation, identify the true underlying model—the holy grail in science. Ideally, this should allow for useful domain knowledge providing a baseline, before the ‘gaps’ are filled in by sophisticated ML techniques, as has previously been done in [8], where the form of a model is specified but not the exact parameters. More broadly, this follows from the theory of combining generative and discriminative techniques, allowing models to learn to describe systems while constrained by example data and broad domain knowledge [9].

Popular approaches in ML for learning models from time-series data derived from dynamical systems include backpropagation through time based recurrent neural networks (RNNs), such as Long Short-Term Memory systems (LSTMs) and Gated Recurrent Units (GRUs). Even though these models have much higher expressive power and the ability to ingest massive amounts of data, several recent works have surprisingly found that echo state networks (ESNs), an older and simpler class of RNN [10] substantially outperform LSTMs in prediction tasks involving chaotic dynamical systems [11,12]. The main historical motivation for ESNs was to avoid the pitfalls of classical deep architectures such as RNNs, namely slow and surprisingly unstable training due to undesirable bifurcations [13] and vanishing/exploding gradients [14]. By contrast, ESNs offer a fast, stable and simple alternative training algorithm via regularized linear regression. ESNs solve a convex optimization problem in closed form, with optimality guarantees. The key disadvantage of ESNs as compared to other RNNs, is the need for augmenting reservoir states with ad-hoc nonlinear combinations to obtain models with good predictive power [3].

In this paper, we present the results of some experiments applying ESNs [2,15] to the prediction of chaotic dynamical systems, and try to gain insight into why they may or may not work in different situations. The benchmark systems used to test our hypothesis are the fully observed Lorenz 63 [16] and the partially observed Lorenz 96 [17] models. We probe ESNs with ablation and perturbation experiments in order to understand their reported successes in the context of Lorenz-based models. Additionally, we provide a simple but rigorous characterization of the deeper reason behind the successes of LSR-ESNs, which leads us to a much simpler surrogate model. Our main contributions can be summarized as follows.

### (a) Main contributions

First, we show in a mathematically rigorous manner that in the regimes where ESNs are successful as surrogates for the Lorenz systems of interest to us, a greatly simplified ESN with an identity reservoir, thus rendering the system feedforward, actually captures all of the essential features of the ESN. We will refer to this simplified method as the *domain-driven regularized regression* (D2R2). Given the alluring possibility of being able to simplify the structure of an ESN without compromising prediction quality which our mathematical development suggests, we performed experiments to explore this opportunity. Specifically, we will take as our starting point two recent papers using ESNs to model dynamical systems [3,11] and the variants used therein. For technical reasons which will be made clear later, the two variants of ESNs from these papers will be referred to as the *low spectral radius* or LSR-ESN [11] and *high spectral radius* or HSR-ESN [3], respectively.

Our experiments show that D2R2 outperforms both the more sophisticated LSR- and HSR-ESN architectures by a notable margin. For example, D2R2 has a mean prediction horizon of 1.60 Model Time Units (MTU) in the context of Lorenz 96, whereas the mean prediction horizon was 10+ MTUs (entire testing trajectory) for Lorenz 63. By contrast, the respective values using LSR-ESN were 1.25 MTUs and 5 MTUs. Thus, D2R2 achieves an improvement of 28% in the L96 case prediction horizon, and greater than or equal to 100% in the Lorenz 63 case. Using FLOP counting, we can show that D2R2 is 496 × more efficient than either form of ESN for Lorenz 96 and 77.7 × more efficient in the context of Lorenz 63.

There are essentially two main reasons for considering the use of machine learned surrogates in the context of predictive modelling of dynamical systems specifically, and in the context of models based on differential equations more generally. The first reason, which is a central theme of the work presented here, is motivated by the cost of computing the model at scale. The second reason is that surrogates may enable feasible solutions in settings where a fundamental understanding or mathematical model of a physical process is lacking, as in vision or autonomous navigation, or even when available data is insufficient to use a principled approach. In this paper, we are focused on both of these goals, with our experiments involving Lorenz 96 representing the second goal, and the precision reduction experiments representing the first.

Energy efficiency as a barrier to scaling has become a major impediment as device densities in supercomputers have grown and the concomitant energy and cooling needs grew alongside to unmanageable levels. In response, *inexact computing* [18,19] has evolved into an attractive prospect, especially in the context of weather prediction [15,20]. While early work [15,21,22] advocated for the use of customized hardware, commercial off-the-shelf processors afforded a more limited set of design choices, notably through word-size or precision [23]. The overarching goal of all of these efforts was to ‘ trade off a *small* amount of quality in the desired solution for disproportionately large savings in energy and execution time’.

In this paper, our second contribution is to explore the role of inexactness through precision and we consider the three standard and commercially available sizes of 64 bits, 32 bits and 16 bits. Again, to our surprise, we discovered that D2R2 provides gains through inexactness with the least amount of degradation in ‘quality’. As a notable example, we show that starting with a prediction horizon of 1.6 MTUs, D2R2 degraded by 13.1% in quality to 1.39 MTUs using 16 bit words for a *factor of four* in savings. For the same change in word size or precision, the better performing ESN (LSR-ESN) degraded from 1.25 MTUs to 0.563 MTUs, representing a degradation of 55.0% in quality. And finally, we show that D2R2 is much more efficient, yielding an improvement of 147% in prediction horizon compared to LSR-ESN at 16 bits of precision.

### (b) Outline of paper

The remainder of this paper is organized as follows: §2 reviews related work in using ML methods for predicting dynamical systems and a brief history of inexactness with an emphasis on its use in dynamical systems and weather prediction. Section 2a discusses previous work on understanding ESNs, and how our approach differs. Section 3 details our ablation studies and other experiments to understand ESN performance. Section 4 is the technical anchor for this paper, and provides a simple yet mathematically rigorous piece of evidence supporting the surprising result that ESNs in the LSR regime are equivalent to a much simpler regularized regression linear model, with *domain-driven* input features/predictors. Section 5 details this simpler model, termed D2R2, and provides comparisons with respect to previous ESN regimes. Finally, in §6 we examine our previous results through the lens of inexactness, evaluating the costs and benefits of using lower-precision approximations in the various models.

## 2. Related work

The idea of using tools from ML to derive the dynamics of physical systems directly from data, is not new [2,5,24–26]. One appeal of a data-driven approach is that effective surrogate models trained on high-fidelity simulation data can be used to accelerate and improve prediction and simulation of complex dynamical systems. Furthermore, for dynamical systems for which equations are unknown, and only observational data are available, models learned from data offer a viable alternative to purely physics-based approximations [7].

Recent advances in deep learning have revived interest in surrogate modelling of complex dynamical systems by providing a variety of new representations and new training paradigms. Earlier studies used deep learning architectures, both feedforward and recurrent, including variants such as LSTMs and GRUs, all of which are trained using the computationally expensive backpropagation through time [4,27] algorithm. However, several recent studies have found that a simpler technique, ESNs, may have comparable to superior performance [3,11]. ESNs are an older technique, developed simultaneously and independently by Herbert Jaeger as ESNs and by Wolfgang Maass as Liquid State Machines [28,29]. In both cases, a large, fixed recurrent reservoir of units (characterized by a sparse, randomly generated recurrence matrix *A*) is driven by an input signal randomly projected through a fixed matrix *W*_in_ into the reservoir state space, while the target is re-constructed by learning a weighted sum of reservoir unit activations.

Training ESNs is significantly faster and cheaper than training an LSTM or other modern RNN using backpropagation through time. ESN performance is known to depend on the Echo State Property, which requires that the influence of the initial conditions of an ESN decays asymptotically to zero with time. An ESN with the Echo State Property, can be proven to be a universal function approximator [30]. Among the classes of ESNs explored within the weather community, one class has the reservoir matrix *A* with LSR (e.g. maximum eigenvalue, low in this case being ≈0.1) [11] which is the LSR regime, while the other is the HSR regime allowing *A*’s with higher spectral radius generally close to 1 [3]. Both ESN types rely on basis function expansion of the reservoir states for good predictive performance.

In addition to the references cited above, the foundational ideas that led to inexactness with emphasis on energy consumption and trading the concomitant cost for quality can be found in [31–34]. Impressive results in using precision as a mechanism for inducing inexactness in weather models have been reported [35–37]. To reiterate, a survey of earlier papers and a broad perspective on inexactness can be found in [1,18] and the reader is referred there to explore additional works that laid the foundation of the field during its early stages of development.

### (a) Understanding ESNs: cracking open the black box

Several previous attempts have been made towards developing formal explanations for the performance of ESN systems [6,10,38–40]. Herbert Jaeger [10] had early on drawn parallels between the structure of an ESN and Taken’s embedding theorem [41], which proves that a sufficient number of previous observations of a dynamical systems forms an embedding with dynamics identical to the original system. However, it has been known in practice that embeddings using the Taken’s formulation are often brittle and of limited utility for real world tasks. Recent work by Eftekhari *et al*. [38] has better characterized ‘stable’ embeddings, which must be geometry preserving. Work by Lu *et al*. [6] uses the concept of generalized synchronization to determine general conditions under which the ESN may provide a good short term (prediction) or longer term (climate) approximation of the target system. Recent and ongoing work by Hart *et al.* [40], closely following the Whitney formulation of Taken’s embedding theorem [42], *almost* proves that an generic ESN is an embedding such that output weights exist that can predict the next step ahead arbitrarily well. However, at present one critical step is incomplete, with the current version only proving that the probability that an ESN mapping is an embedding of an underlying dynamical system is positive. It does not bound this probability, nor does it provide a procedure for selecting key hyperparameters of the ESN algorithm to obtain a stable embedding with high probability. Unfortunately, providing probability bounds may not be of practical utility. Taken’s Theorem, despite provably producing an embedding, is notorious for producing unstable embeddings which are of limited use for prediction. Instead, the goal of our work is to first find a more empirically driven explanation, and second, distil a theoretical understanding from it.

## 3. Experiments for understanding ESNs

**Figure 1.**
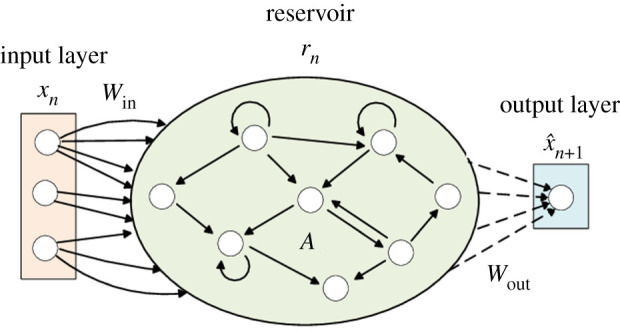
The architecture of an ESN. Inputs $x\in R^{D}$ are fed into the reservoir through input connectivity matrix $W_{\text{in}}\in R^{N\times D}$. The reservoir has hidden state $r\in R^{N}$, and recurrent connections given by $A\in R^{N\times N}$. Output $\hat{y}$ is generated by taking reservoir states *r* multiplied with output connectivity matrix $W_{\text{out}}\in R^{D\times N}$. In autonomous mode, predictions $\hat{x}$ are fed back as inputs to the next time step in order to predict multiple time steps into the future. Note that in an ESN *W*_in_ and *A* are *fixed* matrices—only *W*_out_ is trained. Figure adapted from [43]. (Online version in colour.)

For completeness, we will briefly review the architecture of an ESN first. As shown in figure 1, an ESN is an RNN with a sparsely connected hidden layer called a reservoir. The connectivity and weights *A* of the reservoir neurons are fixed and randomly assigned. The input to the network is projected into the hidden layer by a fixed random mapping *W*_in_.The hidden layer is recurrent and evolves nonlinearly as a function (*σ*) of the previous reservoir state and the current (projected) input. The weights of output neurons *W*_out_ can be adjusted so that the network can reproduce temporal patterns in the input data stream. Typically, the reservoir state is subject to a nonlinear transform *ψ* before being used for prediction. The only weights that are modified during training are the ones that connect the hidden neurons to the output neurons (*W*_out_). *W*_out_ can be computed analytically in closed-form using a convex optimization algorithm.

Overall, ESNs act as a black-box model for modelling dynamical systems, where the system output ${\hat{x}}_{n+1}$ is determined by a combination of a hidden reservoir state *r*_*n*+1_ and input *x*_*n*_. The key difference between ESNs and similar models is that in an ESN, the hidden state is a fixed (non-trainable), random projection function of the input history.

Specifically, we will take as our starting point two recent papers using ESNs to model dynamical systems, [3,11]. Both papers use a nearly identical ESN formulation, which we standardize mathematically as follows:

The reservoir update and prediction functions are given by

3.1 $$\begin{array}{rl} r_{n+1} & =\sigma (Ar_{n}+W_{\text{in}}x_{n})\in R^{N},\;r_{0}=r_{n=0}=0 \end{array}$$

3.2 $$\begin{array}{rl} {\tilde{r}}_{n+1} & =\psi (r_{n+1})\in R^{N} \end{array}$$

3.3 $$\begin{array}{rl} \text{and}\;{\hat{x}}_{n+1} & =W_{\text{out}}{\tilde{r}}_{n+1}\in R^{D}, \end{array}$$

with data dimension *D* and reservoir dimension *N*, where *x*_*n*_ $\in R^{D}$ is the current input data, *W*_in_ $\in R^{N\times D}$ is the *fixed* input projection matrix, *σ*(*u*) : = tanh (*u*) is the reservoir recurrent update function and $\tilde{r}=\psi (r)$ is a simple elementwise nonlinearity, chosen typically to increase the span of the nonlinear features *r*_*n*_. $W_{\text{out}}\in R^{D\times N}$ is a matrix of parameters that linearly combines the elements of ${\tilde{r}}_{n}$ to generate an output prediction ${\hat{x}}_{n+1}$ of the state of the target dynamical system at the next timestep *n* + 1, where the hat denotes an estimate. Note that this ESN is a discrete time system, not a continuous one, although continuous ESNs are also possible and commonly employed [44].

### (a) Training task: teacher forcing

The task for which all ESNs will be trained is to predict the system state at the next time step given the current reservoir state and the ground truth current system state. This is known as *teacher forcing* since intuitively a teacher provides ground truth *x*_*t*_ as the external driving force to the reservoir. The optimization and loss function are

$${\hat{W}}_{\text{out}}:=\underset{W_{\text{out}}}{\mathrm{argmin}}\;\;\ell_{\text{tr}}^{\text{TF}}(\theta_{\text{ESN}};D_{\text{tr}})+\alpha ||W_{\text{out}}|{|}_{2}^{2}$$

and

$$\ell_{\text{tr}}^{\text{TF}}(\theta_{\text{ESN}};D_{\text{tr}}):=\sum_{n=1}^{S_{\text{tr}}}||{\hat{x}}_{n}^{\text{TF}}-x_{n}|{|}_{2}^{2},$$

where superscript TF refers to the Teacher Forcing task. Note that the only ESN parameter trained is *W*_out_, such that *θ*_ESN_ = {*W*_out_}, over the *S*_tr_ training samples from dataset $D_{\text{tr}}:={\left\{(x_{n})\right\}}_{n=1}^{S_{\text{tr}}}$. Note also that the optimization amounts to a simple ridge (linear) regression, where the trade-off between goodness-of-fit and parsimony is controlled by the regularization strength *α*. The size of the reservoir *N* is usually taken to be a few hundred to a few thousand units, depending on the complexity of the target system. After training on some dataset *D*_tr_, we often want to test the performance of our ESN on some testing set *D*_te_. There are two ways to do this evaluation.

### (b) Testing Task 1: teacher forcing

In the teacher forcing (TF) task, a new *r*_*n*_ is generated by replacing input *D*_tr_ with a testing set $D_{\text{te}}:={\left\{(x_{n})\right\}}_{n=q}^{S_{\text{te}}},q<0$. We ‘warm up’ the reservoir, by running several iterations with inputs prior to *x*_1_ (e.g. from *q* to 0), allowing us to have a realistic *r*_0_. After populating the reservoir, we then compare $W_{\text{out}}\tilde{r}$ to *x* from $D_{\text{te}}$. That is, our test/evaluation loss for Task 1 is

$$\ell_{\text{te}}^{\text{TF}}(D_{\text{te}}):=\sum_{n=1}^{S_{\text{te}}}||{\hat{x}}_{n}^{\text{TF}}-x_{n}|{|}_{2}^{2},$$

where *r* is generated from

$$r_{n+1}=\sigma (Ar_{n}+W_{\text{in}}x_{n}),\;r_{n}\in R^{N}.$$

Recall that ${\hat{x}}_{n}^{\text{TF}}$ is computed from ${\tilde{r}}_{n}$, e.g. the nonlinearity *ψ* is still applied. Essentially, this testing task is the same as the training task, except with data set *D*_tr_ replaced with *D*_te_, and with no updating of the *W*_out_ parameter. Note that in every testing step, the system is provided with ground truth data.

### (c) Testing Task 2: student forcing

In contrast to the TF task, in the student forcing (SF) task the input *x*_*n*_ is instead replaced with ${\hat{x}}_{n}$, the predicted output from the previous timestep. This yields an evaluation loss of the form

$$\ell_{\text{te}}^{\text{SF}}(D_{\text{te}}):=\sum_{n=1}^{S_{\text{te}}}||{\hat{x}}_{n}^{\text{SF}}-x_{n}|{|}_{2}^{2},$$

the same as in the TF task. However, the reservoir state *r* in SF mode is generated according to

$$r_{n+1}=\sigma (Ar_{n}+W_{\text{in}}{\hat{x}}_{n}),\;r_{n}\in R^{N},$$

where the Student’s prediction $\hat{x}$ is produced according to

$${\hat{x}}_{n}^{\text{SF}}=W_{\text{out}}\psi (r_{n}).$$

Thus, the SF task only begins with the true testing data in its first time step, and must then correctly predict future time steps using the Student’s own previous predictions. Hence in the SF task prediction errors can *accumulate over time*, rendering it a much more difficult task. This is similar to the task of designing an accurate numerical integrator wherein small but systematic prediction errors can accumulate over time. The key difference is that here we are training a *flexible data-driven* model to predict the next state, as opposed to a principled but rigid theory-driven model.

In summary, we will train our ESN models on the TF task but test them on the TF and, most importantly, SF task.

### (d) Target dynamical systems: ODEs for weather applications

We will primarily test on a pair of ODE systems that emerge naturally in the modelling of weather: Lorenz 63 (L63) [16] and Lorenz 96 (L96) [17]. L63 is a simple three-dimensional system, with dynamics given by

3.4 $$\begin{array}{rl} \frac{\text{d}x}{\text{d}t} & =\sigma (y-x), \end{array}$$

3.5 $$\begin{array}{rl} \frac{\text{d}y}{\text{d}t} & =x(\rho -z)-y \end{array}$$

3.6 $$\begin{array}{rl} \text{and}\;\frac{\text{d}z}{\text{d}t} & =xy-\beta z, \end{array}$$

with parameters *ρ* = 28, *σ* = 10 and $\beta =\frac{8}{3}$ chosen such that the system is in the chaotic regime. For the multi-scale L96 system, the dynamics are governed by

3.7 $$\begin{array}{rl} \frac{\text{d}X_{k}}{\text{d}t} & =X_{k-1}(X_{k+1}-X_{k-2})+F-\frac{hc}{b}\sum_{j}Y_{j,k}, \end{array}$$

3.8 $$\begin{array}{rl} \frac{\text{d}Y_{j,k}}{\text{d}t} & =-cbY_{j+1,k}(Y_{j+2,k}-Y_{j-1,k})-cY_{j,k}+\frac{hc}{b}X_{k}-\frac{he}{d}\sum_{i}Z_{i,j,k} \end{array}$$

3.9 $$\begin{array}{rl} \text{and}\;\frac{\text{d}Z_{i,j,k}}{\text{d}t} & =edZ_{i-1,j,k}(Z_{i+1,j,k}-Z_{i-2,j,k})-geZ_{i,j,k}+\frac{he}{\text{d}}Y_{j,k}, \end{array}$$

where indices *i*, *j*, *k* ∈ [6]. Thus, there are 8 *X*_*k*_, 64 *Y*_*j*,*k*_ and 512 *Z*_*i*,*j*,*k*_ elements. *F* = 20 is a large forcing term, while *b* = *c* = *e* = *d* = *g* = 10 and *h* = 1 are tuned to give appropriate dynamics, such that *X* has a slower timescale than *Y* and *Y* has a slower timescale than *Z*. L96 is interesting because it is frequently used as a prototype system of equations for weather modelling, where *X* represents large-scale weather systems, while *Y* and *Z* represent faster eddies and small-scale convection. Similar comparisons can be made to oceanography or other multiscale physical systems. In many of these systems, the variable of interest is the slower-time variable *X*, while *Y* and *Z* may be unobserved or more difficult to measure. In order to replicate this, when testing on L96 our ESN will have as inputs *x* = *X*, i.e. the 8 dimensional variable, and will not have access to the *Y* or *Z* variables needed to exactly reconstruct *X*’s dynamics. This setting is simultaneously (i) more difficult due to missing observations about *Y* and *Z* and (ii) computationally faster/cheaper since we observe far less information, and thus do not simulate the fine time scale variations of *Y* and *Z*. We start with L63 as it is a simpler test case and can yield deeper understanding, and then we move on to L96 as a more complex and meaningful test case. Data for L63 are generated by using Runge–Kutta 4 (RK4) to integrate forward for 50 000 training steps plus an additional 200 000 testing steps from a starting condition of [1, 1, 1]. We use the same data generation technique for L96 as detailed in [11], taking 500 000 training steps, and an additional 500 000 testing steps (where we take 100 trials, each testing 2000 non-overlapping time steps from the testing steps). In all cases, this meant that the surrogate system (D2R2 or ESN) began with the correct internal state/inputs, and all errors are thus solely due to model deficiencies.

### (e) Experimental details

For L63, ESNs had N=100 units, and *D* = 3. The nonlinearity *ψ* used was odd-squaring, where every other input was squared. *A* was chosen such that every unit had 3 outgoing connections, and then had its spectral radius scaled to *ρ* = 0.1. *W*_in_ was given a block structure such that each reservoir unit received excitation from exactly one input, with connection strength randomly chosen from a uniform distribution U(−σ,σ) where *σ* = 0.5. Reservoir states *r* were initialized to zero (*r*_0_ = 0). Regularization strength was chosen to be *α* = 10^−4^. All experiments were done over 100 trials, with targets taken from the testing-steps regime of the data generation. For the LSR- and HSR-ESNs, this necessitated ‘warming up’ the reservoir for 50 time steps before testing began, by providing true inputs *x*_−50_ to *x*_−1_, before testing on *x*_0_ to *x*_2000_. All code is available online at https://github.com/ankitpatel715/DomainDrivenRegReg.

### (f) Experimental results: exploration, ablation and perturbation studies

ESNs perform surprisingly well in predicting chaotic dynamical systems [3], but why and how remains poorly understood, primarily due to its blackbox nature. In order to elucidate the underlying reasons for ESN performance, we focus on the ESN system with parameters taken from [11], which improved upon the previous state of the art [3], as well as performing exceptionally well on a more difficult multi-scale Lorenz model prediction task with only partial observations of state available. We refer to this ESN as the LSR-ESN, due to having a much smaller spectral radius of *A* than previous (HSR) ESNs. We then performed a series of ablation and perturbation studies, wherein we systematically varied key features of the LSR-ESN system/training parameters, namely *N*, *A*, *W*_in_, *W*_out_, unit activation function *σ*( · ), and nonlinearity *ψ*( · ).

#### (i) Reservoir features resemble inputs

**Figure 2.**
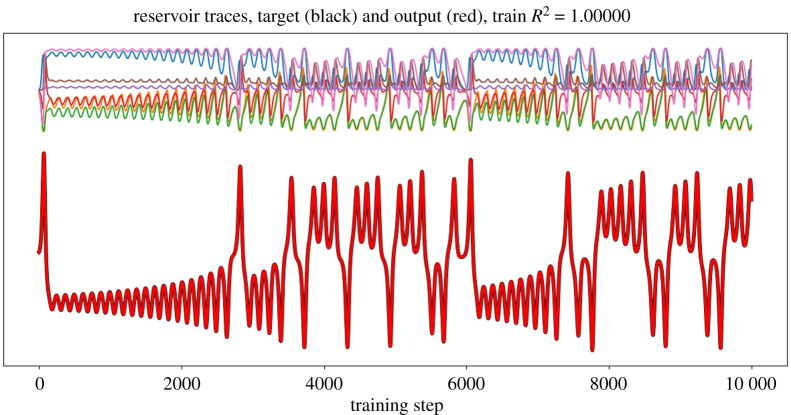
Traces of activations of reservoir nodes over learning time for Lorenz 63. Top: Activation traces $\tilde{r}$ for a set ofrandomly selected reservoir nodes. Bottom: Evolution of the first state component through time (*x*_1,*t*_, black) and reconstruction of that state by linear combination (${\hat{x}}_{1,t}$ computed estimated via *W*_out_) of all the reservoir traces (red). The reconstruction is essentially perfect, with the red line coinciding exactly with the black one. (Online version in colour.)

First, we explore the reservoir features which are linearly combined to generate the prediction. For L63, individual inputs follow an oscillating sinusoidal pattern. Reservoir features follow the same pattern, with matching frequency but phase differences, skewness and/or varying magnitudes, providing useful features for predictions (figure 2).

#### (ii) Reservoir must be sufficiently large for good generalization performance

**Figure 3.**
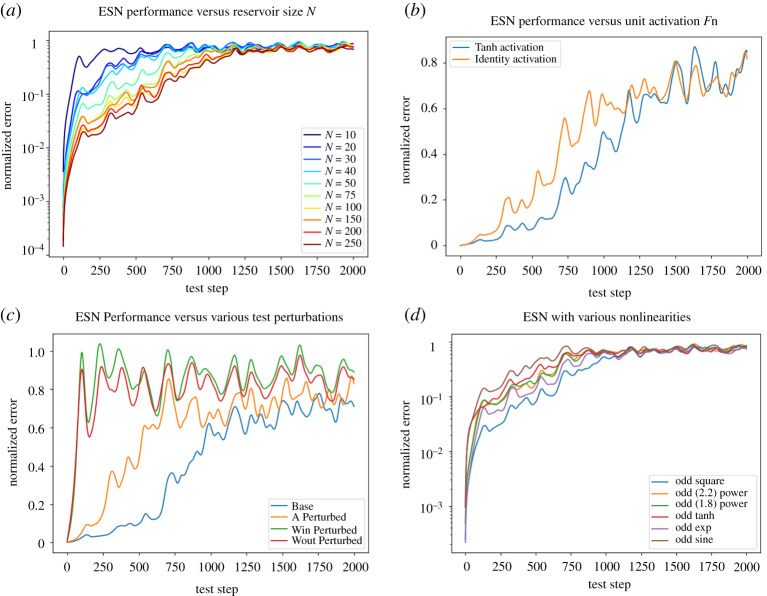
All plots averaged over 100 testing trials. (*a*) ESN performance on the Lorenz63 task as the number of reservoir nodes *N* increases. ESN performance is measured by normalized or relative error in state estimation on the *y*-axis and the autonomous or free prediction horizon the *x*-axis. Once *N* crosses a (dynamical system-dependent) threshold, performance gains asymptote. (*b*) ESN Performance with or without tanh unit activation. Despite canonically being the core nonlinearity that makes ESNs work, it has a limited effect on our task, and removing it actually *increases* performance. (*c*) ESN performance with various testing perturbations. Perturbations to *W*_out_ and *W*_in_ are extremely detrimental, as expected. Surprisingly, perturbations to *A* have a lesser effect, despite changing the effective recurrent nonlinearity applied each time step. (*d*) ESN performance with various feature expansion nonlinearities *ψ*. All changes lead to a minor to moderate performance drop compared to the default ‘odd squaring’—including changing the power from 2 to 1.8 or 2.2. (Online version in colour.)

We begin by evaluating the impact of the reservoir size *N* on the system’s performance on the L63 task. *N* controls the number of features available for the linear regression used to solve for *W*_out_, where the features come from the projected inputs *W*_in_ *x*, further compounded with *A* every update. Thus, we expect that below a critical *N* the span of the features will be insufficient to fit the dynamics at all, with the span of the features saturating with large *N* (as all features originally come from inputs *x*). This is in fact exactly what we see in figure 3*a*: performance quickly improves up until about *N* = 50, and then improvement asymptotes as *N* continues to get larger.

#### (iii) Reservoir unit nonlinearity *σ* is *not* necessary for good generalization

Next, we examine the effect of changing the unit activation function *σ* = tanh which is applied to units at every update. The activation function (compounded throughout each time step) is supposed to provide a critical nonlinearity to the reservoir’s features. This nonlinearity is proposed to be the key reason why ESNs are able to model a wide variety of systems well. However, we discovered that (figure 3*b*), in our parameter regime, removing the nonlinearity entirely had only a minor (but positive) effect on overall performance!

#### (iv) Precision is most critical in *W*_out_ and *W*_in_, least in *A*

For our next test, we consider perturbing the various matrices within the model (*A*, *W*_in_, *W*_out_, *W*_in_) by a small amount during testing (here 1%) , e.g. the perturbations are applied after training. As expected, even small changes/errors in *W*_out_ accumulate over repeated predictions, rapidly decreasing the accuracy. Changes to *W*_in_ have a similar effect. Surprisingly, perturbing *A* has a relatively smaller effect (compared to others perturbations) (figure 3*c*), despite the commonplace intuition that the reservoir’s recurrent nonlinear update should provide useful features for prediction.

#### (v) Feature expansion nonlinearity *ψ* is critical

One non-standard part in many of the recent ESNs [3,11] (compared to the original ESN/LSTM) formulation, is the additional nonlinearity *ψ*, which is applied to the reservoir unit activations before they are used to make predictions. Both [3,11] used a nonlinearity which implements ‘odd squaring’, where every other element is squared (even elements remain unchanged). Both papers note that this extra nonlinearity *ψ* is critical to their final performance on their test cases. We consider various perturbations and choices for this *ψ*, from changing the order of the odd polynomial (to 1.8 or 2.2), as well as changing the odd function to be a tanh, exp or sin rather than a quadratic (figure 3*d*). Nearly every considered change moderately degraded performance (with odd exponential being the least degraded). Most surprisingly, even the variations in the power (e.g. taking a 1.8 or 2.2 power) lead to notable drops in performance.

#### (vi) A reservoir-free ESN achieves nearly identical performance

**Figure 4.**
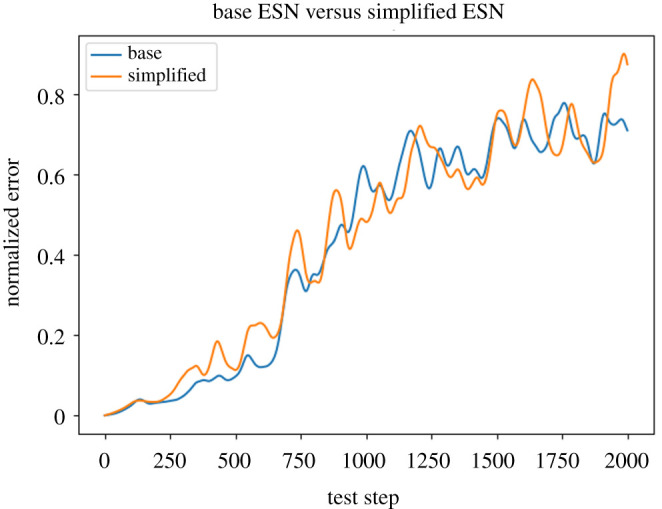
ESN Performance compared to ‘simple’ ESN, averaged over 100 testing trials. Despite removing both *A* and the tanh nonlinearity (e.g. the main components that typically drive reservoir nonlinearity), performance remains extremely similar. (Online version in colour.)

Taken together, the surprising results above suggest that we try a simplified ESN model, in which the unit nonlinearity *σ* = tanh is changed to identity (i.e. removed), and the adjacency matrix *A* is removed entirely, i.e. *A* = 0 and *σ*(*r*) = *r*. For this setting, we also had to change the *W*_in_ matrix from block structure to fully dense in order to get decent performance. Surprisingly, this drastically simplified LSR-ESN had performance that is *virtually indistinguishable* from the state of the art in [11] (figure 4)! This is despite removing both the unit nonlinearity and the reservoir recurrence matrix, which are typically thought to be essential components in an ESN.

## 4. Empirical observations distilled: LSR-ESN simplifies to polynomial regression

The previous experiments yield interesting insights into the LSR-ESN model. To summarize, the reservoir size *N* is fairly inconsequential once it exceeds a critical number *N*_*c*_ of units, endowing the model with sufficient capacity. Changes to *W*_in_ and *A* have a relatively minor effect, while the system is significantly more sensitive to changes in *W*_out_. The tanh nonlinearity is of relatively low importance, and can even be replaced with the identity function. In stark contrast, the precise form of *ψ* is critical: even small changes away from odd-squaring such as odd-exponential (*ψ*(*r*) = exp (*r*)) or odd-2.1 power (*ψ*(*r*) = *r*^2.1^) result in dramatic failure. Surprisingly, the hyperparameter configuration that performs best on L63 is *A* = 0 with no reservoir unit nonlinearity, i.e. *σ*(*r*) = *r*.

How can we explain this surprising result? We can gain some intuition by substituting these conditions into the original ESN equation (3.1), resulting in a reservoir-free ESN of the form

4.1 $$\begin{array}{rl} & r_{n+1}=W_{\text{in}}x_{n}\\ \text{and}\; & {\hat{x}}_{n+1}=W_{\text{out}}{\tilde{r}}_{n+1}=W_{\text{out}}\psi (W_{\text{in}}x_{n}). \end{array}\}$$

In this special case, the ESN model reduces to a (ridge) linear regression model with *nonlinear input features ψ*(*W*_in_*x*). Since the nonlinearity *ψ* is the odd-squaring operation (*ψ*(*r*_*i*_) = *r*_*i*_ if *i* is even and $\psi (r_{i})=r_{i}^{2}$ if *i* is odd), these are *quadratic polynomial* input features. Note that in the teacher forcing task, the nonlinearity *ψ* does not get iterated over time in the ESN dynamics because it is only used to generate the prediction ${\hat{x}}_{n+1}$ but this prediction is not the next input *x*_*n*_. In contrast, in the student forcing task, the next input is $x_{n+1}:={\hat{x}}_{n+1}$ and so in this case *ψ* would iterate. However, note that in this paper we only train the ESN on the teacher forcing task. (We test it on the student forcing task.)

More generally, for the LSR-ESNs we used from [3,11], we cannot ignore the dependence on *A* as *A* ≠ 0. But intuitively the LSR condition $\rho (A):=||A|{|}_{*}\approx 0.1<1$ implies that the dependence of the reservoir state *r*_*k*_ on past inputs *x*_*k*−*τ*_ decays exponentially quickly in lag time *τ*, i.e. scaling as *ρ*(*A*)^*τ*^. Thus the reservoir has a very short memory, or equivalently, the influence of each new input sample *x*_*k*_ decays exponentially quickly with half-life *τ*_1/2_ : = −ln2/ln*ρ*(*A*). This result is formalized in the following Lemma.

**Lemma 4.1.**

*Let E be an ESN with reservoir activation function set to identity σ*(*r*) = *r*. *Consider the regime where the spectral radius of the reservoir recurrence matrix A is low, i.e*. $\rho (A)=||A|{|}_{2}\ll 1$. *Then the reservoir state of E at time T* ***for the teacher forcing task*** *can be written as a series*

$$r_{t}=\sum_{\tau =1}^{t}A^{\tau -1}W_{\text{in}}x_{t-\tau }+A^{t}r_{0},$$

*and the operator norm of the input–output sensitivity matrix* $S_{t,\tau }:=\partial r_{t}/\partial x_{t-\tau }\in R^{N\times N}$ *decays exponentially quickly in the spectral radius of A*:

$$\rho (S_{t,\tau })\leq \rho {\left(A\right)}^{\tau -1}\rho (W_{\text{in}}).$$

**Proof.**

Substituting *σ*(*r*) = *r* into the definition of an ESN (equation (3.1)) yields a linear dynamical system of the form

$$r_{k+1}=Ar_{k}+W_{\text{in}}x_{k},\;\forall \;k\in N_{+}.$$

Iterating this recurrence relation gives us the series form for *r*_*t*_ above, as desired. As mentioned above, in the teacher forcing task *ψ* only affects the prediction for ${\hat{x}}_{k+1}$ but not the reservoir state (see equation (3.1)). Taking a gradient with respect to *x*_*t*−*τ*_ yields input-output sensitivity *S*_*t*,*τ*_ = *A*^*τ*−1^ *W*_in_. Taking the operator norm $\rho (\cdot )=||\cdot |{|}_{2}$ of both sides and invoking the fact that matrix norms induced by vector norms are submultiplicative (||AB||≤||A||||B||) yields the desired result. ▪

Note that applying this lemma to the reservoir-free case *A* = 0 immediately yields the earlier result above:

**Corollary 4.2.**

*Let E be an ESN with no reservoir i.e. A* = 0. *Then the reservoir state dynamics and output predictions for E reduce to equation* (4.1).

This simplification explains why *either W*_in_ being dense or *A* being non-zero helped performance: either condition alone was sufficient to *mix the parameters*, allowing for multiple polynomial terms of various orders which greatly increase predictive power. Even if the spectral radius of *A* is non-zero, small spectral radii may perform similar to the *ρ*(*A*) = 0 case. In order to test this potential explanation, we fit the L63 and L96 systems directly using regularized polynomial regression and compare their performance to that of ESNs.

## 5. Regularized regression with domain-driven features

### (a) Experimental details

For L96, LSR-ESN had N=1500 units, and *D* = 8. The nonlinearity *ψ* used was odd-multiplication of previous two inputs, where every other input *x*(*k*) was replaced with *x*(*k* − 1) · *x*(*k* − 2). *A* was chosen sparsely such that every unit had three outgoing connections, and then had its spectral radius scaled to *ρ* = 0.1. *W*_in_ was given a block structure such that each reservoir unit received excitation from exactly one input, with connection strength randomly chosen to be sampled from a uniform distribution U(−σ,σ) where *σ* = 0.5. Reservoir states *r* were initialized such that *r*_0_ = 0 and the regularization strength was chosen to be *α* = 10^−4^. Note that 200 testing steps is equal to one Model Time Unit (MTU), a standard measure in L96, and thus we measure divergence times in MTUs, not testing steps, where divergence time is defined when normalized error exceeds 0.3 (a standard L96 metric).

We also compared to the previous [3] HSR-ESN, with the following changes: the sparsity of *A* was set to 6, spectral radius *ρ*(*A*) = 1.2, and *W*_in_ connection strength *σ* = 0.1.

### (b) Regularized regression with low-degree polynomial features

**Figure 5.**
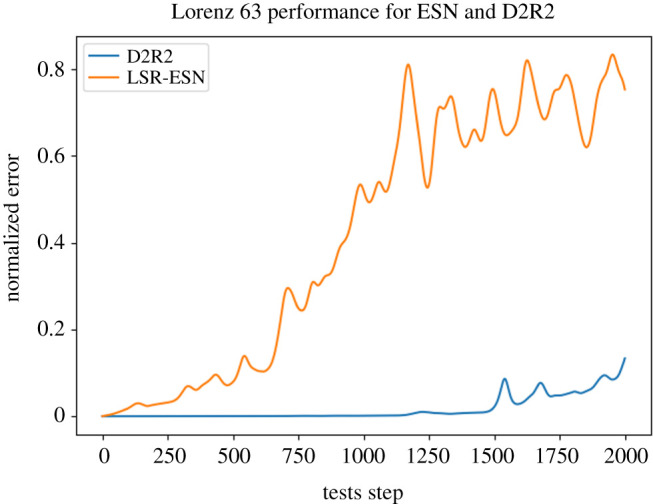
ESN Performance compared to D2R2, averaged over 100 trials. As is expected in this case (where the true update is in the span of polynomial features), D2R2 performs near optimally. (Online version in colour.)

For the D2R2, we directly solve for *W*_out_ as

$$W_{\text{out}}=\underset{W}{\mathrm{argmin}}||Wg(x_{n-1})-x_{n}||+\alpha {||W||}_{2}^{2}$$

e.g. ${\hat{x}}_{n}=W_{\text{out}}g(x_{n-1})$, where *g* is a function that that performs basis function expansion of *x*_*n*−1_. For polynomial regression, *g* : = *g*_*m*_ returns all polynomial terms up to a specified order *m*, e.g. *g*_2_({*x*, *y*}) = {1, *x*, *y*, *x*^2^, *y*^2^, *xy*}. Unsurprisingly, this method works exceptionally well on L63 for orders between 2 and 4, as Lorenz (equation (3.4)) has a quadratic right-hand side update equation (yielding a quadratic if updated with forward Euler, and a quartic if solved via RK4). D2R2 has performance notably superior to the original LSR-ESN ESN [11] (figure 5).

**Figure 6.**
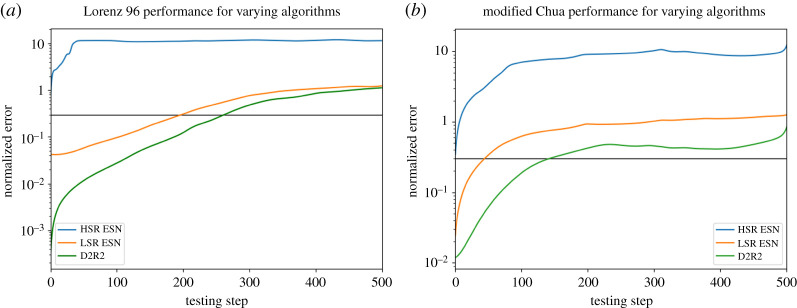
Both plots are averages over 100 trials. (*a*) Lorenz 96 performance across varying algorithms. The direct polynomial regression outperforms both types of ESNs. The horizontal line at 0.3 is used as a standard measure of error for L96 to determine when a surrogate fails. (*b*) Modified Chua Task Performance across varying algorithms. The HSR ESN fails across most training trials due to the model being in a different part of state-space than it was trained on. LSR ESN and D2R2 both do better, with D2R2 being best overall. (Online version in colour.)

We obtain similar results for L96 (equation (3.7)). Despite the true update for *X* not being available (as it depends on terms from *Y* and *Z* which are not available), D2R2 (order 4) does quite well, taking 50% longer to reach a relative error of 0.3 (figure 6). Thus, it is clear that D2R2 serves as an upper bound for the LSR-ESN performance, providing more evidence that in this regime the ESN is just performing a noisy version of direct polynomial regression. However, it is quite clear that the original ESNs (from now on, HSR-ESN) [3] are doing something quite different. With a higher spectral radius, less critically depending on the nonlinearity *ψ*, it clearly is not doing a polynomial regression on expanded reservoir activations. Nevertheless, D2R2 is superior in the L63 case (where the exact solution is known and available, not shown), and also in the L96 case (where the exact solution is not available in the feature span). D2R2 also compares well with previous approaches [4].

What about more complicated systems, i.e. those that have nonlinearities that are not polynomial? We run an additional test using the modified Chua attractor, which has a mix of linear and trigonometric terms and is significantly harder to fit, even for D2R2 (now modified so that *g* also returns trigonometric terms) figure 6*b*. Nevertheless, D2R2 still does better than an LSR-ESN (with an odd sine nonlinearity *f*) or a HSR-ESN. Note that the HSR-ESN did *not* need to have its internal nonlinearity changed from odd squaring, although the LSR-ESN did require *f* to be changed in order to work, suggesting the HSR-ESN may be more valuable in cases where the family of the true dynamical system is unknown. However, if the family is known, D2R2 may be a viable alternative in terms of speed, generalization accuracy and interpretability.

Finally, we did a larger scale study of performance of the three algorithms on L96, with 100 different testing sets considered for each model. 99 of the 100 testing sets were not (temporally) adjacent to the training set $D_{\text{tr}}$, which provided a harder test of whether the surrogate system had truly learned the target L96. LSR-ESN did well, but the D2R2 outperformed it in every testing metric. The HSR-ESN did fairly poorly, mostly because approximately half the time it failed to generate a useful prediction trajectory, instead immediately failing, showing that the HSR-ESN has difficulty generalizing to novel training data $D_{\text{te}}$ (that still comes from the same L96 system). Even when it did work, it still performed inferior to our D2R2 (table 1).

**Table 1.** Performance metrics for the three algorithms (L96). There was one large training set *D*_tr_, and then 100 smaller training sets *D*_te_ for each algorithm. For L96 tasks, the prediction is considered diverged when normalized testing error (SF) exceeds 0.3. Time until divergence is measured in model time units (MTU, 1*MTU* = 200Δ*t*), a standardized timescale for L96. The HSR-ESN only worked on a subset of testing cases that were temporally distant from generated training data, while others instantly failed. For this and future tables, results are given as mean ± s.d. over 100 trials.

	training error	TF error	SF error	divergence time
LSR-ESN	1.80 × 10^−4^	4.33×10−2±1.75×10−3∫	1.16 ± 1.06 × 10^−1^	1.25 ± 4.85 × 10^−1^
HSR-ESN	7.10 × 10^−5^	4.08 × 10^−1^ ± 3.37 × 10^−1^	1.09 × 10^1^ ± 8.62	4.53 × 10^−1^ ± 5.93 × 10^−1^
D2R2	2.24 × 10^−4^	2.28 × 10^−4^ ± 2.11 × 10^−5^	1.13 ± 1.06 × 10^−1^	1.60 ± 5.31 × 10^−1^

### (c) Asymptotic complexity analysis as a measure of cost

As a final way of comparing methods, we consider an analysis of the complexity of each of our methods in the context of both the L63 and L96 systems when using LSR-ESN, HSR-ESN and D2R2. For an ESN model where LSR or HSR will have the same asymptotic cost, one step of inference is given by

$${\hat{x}}_{n+1}:=\hat{x}(t_{n+1})=W_{\text{out}}\psi (\sigma (Ar_{n}+W_{\text{in}}{\hat{x}}_{n})),\;\forall \;n\in N_{+}$$

Recall that here, we have previous state *r*_*n*_, previous output estimate ${\hat{x}}_{n}$, *N* by *N* adjacency matrix *A*, and *N* × *d* dimensional *W*_in_ and *W*_out_ and output nonlinearity *ψ*. Following conventions used in asymptotic analysis of algorithms, floating point add, multiply or tanh take *O*(1) time. Then, the number of operations is ((2*N*^2^ − *N*) + (4*Nd* − *d* − *N*) + 2*N*), where the *N*^2^ term is due to the multiplication *A* × *r*_*n*_ of an (*N* × *N*) matrix with a (*N* × 1) vector, The *Nd* terms are due to the multiplications with *W*_in_ and *W*_out_ where each is the product of a single (*N* × *d*) matrix with a *d* dimensional vector. For our ESN tasks, *N* ≫ *d*, so the overall asymptotic complexity is *O*(*N*^2^), with an exact cost of 4,547,992 FLOPs per prediction step for L96 (with *N* = 1500, *d* = 8), and 21 197 FLOPs per prediction step for L63 (*N* = 100, *d* = 3).

By contrast, recall that D2R2 with *ψ* = *g*_*m*_ may have a different number of nonlinear transformations *N*_2_, each with their own cost. For our case of the polynomial features up to order *m*, the number of features of *g*_*m*_ will be *N*_2_ = $\sum_{i=0}^{m}((d+i-1)!)/(i!(d-1)!)$. The final inference cost is therefore *O*(*N*_2_ *d*) = *O*(*d* (*d* + *d*^2^ + · · · + *d*^*m*^)) = *O*(*d*^*m*+1^). This can be reduced further if more information is known about the features. The cost of *O*(*d*^*m*+1^) above accounts for *every* polynomial combination for orders up to *m* in the spirit of worst-case analysis. More precisely, for our L96 experiment we use up to quartic (*m* = 4) terms, with a *d* = 8 dimensional input. Thus, calculating the features takes 0 + 8*0 + 36*1 + 120*2 + 330*3 FLOPs, while the final output multiplication takes an additional 2(1 + 8 + 36 + 120 + 330)*(8) − 8), for a total of 9178 FLOPs per prediction step. Similarly, for our L63 experiment we also use a quartic (*m* = 4), with *d* = 3, so calculating features takes 0 + 3*0 + 6*1 + 10*2 + 15*3 FLOPs, and the final output takes an additional 2(35)*(3) − 3, for a total of 278 FLOPs per prediction step.

We compare both exact and asymptotic costs for specific experiments performed in the context of *L*63 and *L*96, where *N* = 100, *N*_2_ = 35 and *d* = 3 in the former case, whereas *N* = 1500, *N*_2_ = 495 and *d* = 8 in the latter. Note however that *m* = 4 in both cases. As summarized in table 2, we note that for these specific parameters, using required FLOPs as our metric, D2R2 is a factor of 77.7 times more efficient than an ESN in the context of L63, whereas when considering L96, it is a factor of 496 times more efficient. Note that the asymptotic cost tends to underestimate the cost of the ESNS and overestimate that of D2R2!

**Table 2.** Complexity estimates of the ESN and D2R2 for our Lorenz family of systems, both asymptotic and exact, in terms of FLOPs per testing iteration.

	L63 estimate	L63 exact	L96 estimate	L96 exact
ESN: *O*(*N*^2^)	1 × 10^4^	2.12 × 10^4^	2.25 × 10^6^	4.55 × 10^6^
D2R2: *O*(*d*^*m*+1^)	2.43 × 10^2^	2.73 × 10^2^	3.28 × 10^4^	9.18 × 10^3^

We are also currently completing a companion paper with detailed modelling and measurement-based validations of these findings. There we employ realistic machine models [23,45] and measurement counters that are built into modern processors.

## 6. Inexactness through precision variation

Recall that our goal here is to consider the efficacy of different ML models and their ability to serve as surrogates in the context of dynamical systems. In keeping with the analysis in the previous section, our next goal is to understand how much of the cost of a system can be lowered through inexactness, while quality—defined to be the prediction accuracy—is maintained at acceptable levels. There has been significant work done in understanding the role of inexactness in the context of trading cost for quality in the Lorenz 96 system directly [23,46–49]. To reiterate, our goal in this section is to further understand this in the context of how such an approach will yield gains or savings in the context of machine learned surrogates. Using commercial-off-the-shelf word sizes, we will explore double (64 bits), single (32 bits) and half (16 bits) precision values in our analysis below. For the precision reduction experiments, we used standard software emulation from the python numpy library to reduce the specified precision from double (64 bit) to single (32 bit) or half (16 bit)—thus we cannot directly measure walltime speed-ups, we can only estimate savings.

Our ‘knob’ is precision, that is we control the amount of bit precision available to the different components of a model (*A*, *W*_in_, *W*_out_) and as before, we will be considering LSR-ESN, HSR-ESN and D2R2. Since training can be viewed as a cost that is an initial investment which can then be amortized over a multitude of predictive inference instances, our focus will be not to alter training but rather consider precision variations during the prediction or inference phase. Continuing, we will consider three different methodologies of lowering precision. First, the most obvious method is to lower the precision of all variables used during inference. Next, guided by our results from figure 3*d* showing that *W*_out_ has the largest perturbation sensitivity, we will consider lowering the precision of *W*_out_ alone. Finally, we will, in contrast with this second case, lower the precision of all variables *except* for *W*_out_. We note that lowering precision from 64 to 32 bits had no effect on the quality of the solution, while lowering precision down to 16 bits did. Notably, the impact in the latter case was more pronounced for HSR-ESNs compared to LSR-ESNs.

### (a) Exploiting inexactness in the entire model

**Figure 7.**
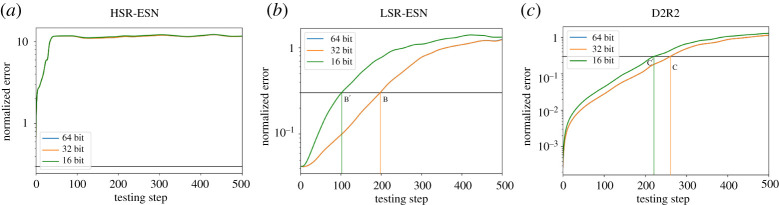
ESN quality (L96 task) with error on the ordinate axis derived through inexactness where all variables in the respective systems are lowered in precision. HSR-ESN (*a*), LSR-ESN (*b*), D2R2 (*c*), where points marked (A,A’) (B,B’), (C,C’) show where the 0.3 mean error threshold is crossed for the (64 and 32 bit, 16 bit) version. Note that the 64 bit and 32 bit lines and crossing points are indistinguishable, so they are marked with the same label, and that A and A’ (HSR) are at *t* = 0. (Online version in colour.)

Let us consider (figure 7) where we illustrate the effect of lowering precision from 32 bits down to 16 bits on prediction quality. D2R2 had the best performance at all precision levels. As shown in figure 7, the *divergence time*—the time at which the normalized relative prediction error exceeds 0.3—is labelled by points (A, A’), ( B, B’) and (C, C’) (mean ± s.d. shown). At 32 bits precision, the divergence time as shown in table 3 has a mean of 1.60 MTU for D2R2 whereas it is 1.24 MTU for LSR-ESN and 0.453 MTU for HSR-ESN. Also, as shown in table 4, dropping precision further down to 16 bits degraded D2R2 less than 15% in prediction quality, while LSR-ESN and HSR-ESN, respectively, degraded by 55% and 76%. Thus beyond the gains reported in the previous section, for 13.1% degradation at 16 bits which is competitive in quality (12% better) with LSR-ESN at 32 bits, D2R2 is more efficient by an additional multiplicative factor of 2.

**Table 3.** (L96) Details of comparing all three methods at 32 bits of precision where the divergence time in the last column is the main point of conclusion.

	training error	TF error	SF error	divergence time
LSR-ESN	1.80 × 10^−4^	4.32 × 10^−2^ ± 1.75 × 10^−3^	1.18 ± 9.81 × 10^−2^	1.24 ± 4.74 × 10^−1^
HSR-ESN	7.07 × 10^−5^	4.08 × 10^−1^ ± 3.37 × 10^−1^	1.09 × 10^1^ ± 8.61	4.53 × 10^−1^ ± 5.93 × 10^−1^
D2R2	2.24 × 10^−4^	2.28 × 10^−4^ ± 2.11 × 10^−5^	1.14 ± 1.06 × 10^−1^	1.60 ± 5.31 × 10^−1^

**Table 4.** (L96) Comparing the three methods with 16 bits of precision.

	training error	TF error	SF error	divergence time
LSR-ESN	6.70 × 10^−4^	4.32 × 10^−2^ ± 1.75 × 10^−3^	1.29 ± 8.58 × 10^−2^	5.63 × 10^−1^ ± 2.20 × 10^−1^
HSR-ESN	9.87 × 10^−4^	4.09 × 10^−1^ ± 3.37 × 10^−1^	1.10 × 10^1^ ± 8.56	1.06 × 10^−1^ ± 1.29 × 10^−1^
D2R2	4.23 × 10^−4^	3.78 × 10^−4^ ± 1.70 × 10^−5^	1.16 ± 9.95 × 10^−2^	1.39 ± 5.14 × 10^−1^

**Table 5.** Performance (L96) metrics for the three algorithms, 32 bits *W*_out_. Details the same as the previous overall comparison. Once again, HSR-ESN fails a portion of trials, making its average time to cross 0.3 error highly variable.

	training error	TF error	SF error	divergence time
LSR-ESN	1.80 × 10^−4^	4.32 × 10^−2^ ± 1.75 × 10^−3^	1.17 ± 1.07 × 10^−1^	1.24 ± 4.75 × 10^−1^
HSR-ESN	7.10 × 10^−5^	4.08 × 10^−1^ ± 3.37 × 10^−1^	1.09 × 101 ± 8.62	4.53 × 10^−1^ ± 5.93 × 10^−1^
polynomial fit	2.24 × 10^−4^	2.28 × 10^−4^ ± 2.11 × 10^−5^	1.14 ± 1.00 × 10^−1^	1.60 ± 5.31 × 10^−1^

**Table 6.** Performance (L96) metrics for the three algorithms, 16 bit *W*_out_. Details the same as the previous overall comparison. Once again, HSR-ESN fails a portion of trials.

	training error	TF error	SF error	divergence time
LSR-ESN	4.42 × 10^−4^	4.32 × 10^−2^ ± 1.75 × 10^−3^	1.24 ± 8.42 × 10^−2^	7.47 × 10^−1^ ± 2.72 × 10^−1^
HSR-ESN	5.32 × 10^−4^	4.09 × 10^−1^ ± 3.37 × 10^−1^	1.10 × 101 ± 8.57	1.36 × 10^−1^ ± 1.68 × 10^−1^
polynomial fit	3.24 × 10^−4^	3.26 × 10^−4^ ± 1.86 × 10^−5^	1.16 ± 1.01 × 10^−1^	1.39 ± 5.21 × 10^−1^

We remark in passing that HSR-ESN failed during some training trials at both precision values. In order to better understand these differences, we next test the case of only reducing the precision of *W*_out_, which, to reiterate, had the most impact on the output.

### (b) Understanding the impact of inexactness on *W*_out_ and prediction quality

**Figure 8.**
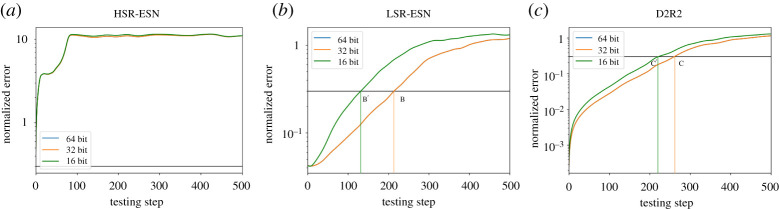
ESN Performance (L96 task) w/ inexactness in *W*_out_ across varying algorithms. (*a*) HSR-ESN, (*b*) LSR-ESN, (*c*) D2R2. In all cases, dropping only *W*_out_ to 32 bit had no discernible effect. Details same as previous figure. (Online version in colour.)

We now consider the case where only the variable *W*_out_ has its precision lowered. In this case, the precision of *A* and *W*_in_ are unchanged, meaning that the recurrent updates of the ESN methods will be significantly less affected. The D2R2 in figure 8 is essentially unchanged, showing that *W*_out_ is the key term in D2R2. Looking at tables 5 and 6, we see that D2R2 is very similar in prediction quality when compared to the previous case where all the variables were lowered in precision, while both ESNs have much better prediction quality when *W*_in_ and *A* are left untouched. For example, with 16 bits of precision, the mean divergence time for LSR-ESN in this case is 0.747 MTUs, whereas it is 0.563 MTUs when all precisions were lowered (tables 4 and 6). This confirms our observation that D2R2 is primarily sensitive to *W*_out_, while the ESNs also have a greater dependence on *A* or *W*_in_

### (c) Prediction quality by preserving *W*_out_

Finally, we consider the opposite case where all variables but *W*_out_ have their precision reduced. This revealed some interesting behaviour. Since *W*_out_ plays a dominant role in the D2R2, lowering precision in other parts of the system has hardly any effect, which can be seen both visually (not shown) and in the tables 7 and 8. However, in the case of ESNs, lowering the precision of *A* and *W*_in_ can still have a large effect (tables 7 and 8)! The ESNs sees a moderate decline, similar to their performance in the case of reducing all variable precision.

**Table 7.** Performance (L96) metrics for the three algorithms for this case with 32 bits of precision in all variables but *W*_out_.

	training error	TF error	SF error	divergence time
LSR-ESN	1.80 × 10^−4^	4.32 × 10^−2^ ± 1.75 × 10^−3^	1.17 ± 1.07 × 10^−1^	1.24 ± 4.77 × 10^−1^
HSR-ESN	7.10 × 10^−5^	4.082 × 10^−1^ ± 3.37 × 10^−1^	1.09 × 10^1^ ± 8.61	4.54 × 10^−1^ ± 5.94 × 10^−1^
polynomial fit	2.24 × 10^−4^	2.28 × 10^−4^ ± 2.11 × 10^−5^	1.13 ± 1.07 × 10^−1^	1.60 ± 5.31 × 10^−1^

**Table 8.** Performance (L96) metrics for the three algorithms with 16 bits of precision in all variables but *W*_out_.

	training error	TF error	SF error	divergence time
LSR-ESN	4.95 × 10^−4^	4.33×10−2∫±1.75×10−3	1.27 ± 8.87 × 10^−2^	5.99 × 10^−1^ ± 2.42 × 10^−1^
HSR-ESN	8.19 × 10^−4^	4.08 × 10^−1^ ± 3.37 × 10^−1^	1.09 × 10^1^ ± 8.61	2.01 × 10^−1^ ± 2.57 × 10^−1^
polynomial fit	3.03 × 10^−4^	2.28 × 10^−4^ ± 2.11 × 10^−5^	1.13 ± 1.07 × 10^−1^	1.60 ± 5.31 × 10^−1^

Our results for this section show that the ESNs, in addition to being more expensive due to their dependence on recurrence through *A* and *W*_in_, also require these variables to be relatively high precision in order to maintain their performance. By contrast, D2R2 depends primarily on *W*_out_, and can better tolerate low precision (e.g. down to 16 bits) than other methods and is therefore a very good candidate for exploiting inexactness.

Stepping back, our results suggest that employing the approach of inexactness could yield significant savings in computational cost using D2R2 while producing acceptable accuracy.

## 7. Conclusion, limitations and future directions

ESNs and other ML techniques have been increasingly used to model dynamical systems, with the goal of using these for tasks of real-world importance such as weather modelling or simulation of physical systems. However, our experiments clearly reveal that the best performing ESN [11] on the Lorenz 96 benchmark effectively does a direct polynomial regression in an implicit and more computationally costly manner. This speaks to the importance of systematically analysing and interpreting the performance of ‘blackbox’ models by executing sensitivity analyses and ablation studies.

We find that the D2R2, which is quite similar to the data-driven system identification model SINDy, a technique known to scale up to fluid flow simulations, is cheaper and faster than a traditional ESN, with performance that is comparable or better. Thus, it may be that, for a wide variety of physical dynamical systems, a simple linear regression model, with an appropriate choice of domain-driven input features and expansion operator, may be superior (in both performance *and* cost) to a state-of-the-art deep learning system. Of course, this technique is only implementable when the form of the dynamics is at least partially known, such as the functional form of the PDEs or ODEs (if not their exact parameters), something that may be known in practice for many physical systems.

We argue that a significant lesson learned here is that using knowledge about the underlying system, typically from the domain of physics-informed mathematical models, can help significantly with scaling. Thus knowing the structure of Lorenz 96 was key to deriving D2R2 and therefore, can play an equally important role in general in innovating machine learned surrogate models for ODE and PDE based multi-scale systems like weather prediction.

Building on this line of thinking further, the power of using domain knowledge to compensate for and perhaps overtake features derived from backpropagation in RNNs is an intriguing possibility. Common wisdom dictates that once the cost hurdles to training with backpropagation are overcome, then the resulting models should be more powerful than simpler approaches such as ESNs, originally created to cope with training costs. However, much to our surprise, our findings here suggest that returning to simpler models such as ESNs and D2R2, augmented/guided by principled domain knowledge, can outperform methods based on backpropagation. A systematic study of this hypothesis is warranted, especially in the context of physics-informed multiscale mathematical models.

Surprisingly, we also showed the value of the simplification that D2R2 afforded over canonical ESN solutions, in providing reductions in cost with little to no reduction in prediction quality. Our next goal is to validate the gains claimed in this paper using detailed measurement methods of energy and time at the hardware level [50]. Furthermore, significant additional reductions in cost have been reported by judicious reinvestment of the savings in energy (and time) gleaned through inexactness [45]. Such reinvestment strategies are also a significant direction worth pursuing, and here we believe ensemble models will be a natural vehicle to explore.

*Limitations and Future Work*. This paper only dealt with relatively simple systems of ODEs: The L63, L96, and modified Chua attractors. Although these capture many of the critical properties of real-world weather dynamics (chaos, multiscale system (L96)), they are far simpler than a shallow water model, for example.

The D2R2 model presented here relies on domain knowledge in a critical way: the true model needs to be within the span of the domain-driven features. For more realistic weather models with much greater complexity, and unknown equations of motions, this is an unrealistic assumption. Instead, we propose that the basic formulation, wherein one combines knowledge-driven and data-driven features, should still be valid. Such an approach would combine the benefits of both strategies and leverage the massive amounts of data available today. In future work, we plan to develop and apply this technique to more complex and realistic weather models, for example, a shallow-water model. Note that here SINDy-style models have been successful [51], and so a hybrid D2R2 approach should perform at least as well if not better.

Another interesting and important direction would be to develop a deeper understanding of the role of each bit of precision in the surrogate model. Despite their importance, the nature and quality of approximations induced by inexact (variable precision) methods remain poorly understood. The inexact approach employed in this paper (the ablation study to determine the most important bits in *W*_out_) is in a certain sense ad hoc: the impact of additional bits of numerical precision in such simulations is unclear. A rigorous characterization would provide answers to several natural questions.

— How does pruning [21] or reducing bit precision impact the nature of the approximate dynamics and the quality of the computed solution?
— How does precision impact approximation quality and performance when used in conjunction with machine-learned surrogate models (e.g. neural networks)?
— How does the impact of a adding a bit to the state representation differ from a adding a bit to the parameter representation in an ODE/PDE system? How should a computational scientist adjudicate between the two alternatives?

Answering these questions, with help from recent advances in understanding the representation of neural networks [52–54], would enable us to develop an principled bit allocation scheme, specialized for the domain of weather modelling.

## Data accessibility

This article has no additional data.
